# New Onset of Seizures Leading to the Delayed Diagnosis of Sturge-Weber Syndrome in Adulthood: A Case Report

**DOI:** 10.7759/cureus.88825

**Published:** 2025-07-26

**Authors:** Mumin Idris, Muskaan Bhagchandani, Kiran Kumar, Elmunzer A Ibrahim

**Affiliations:** 1 Internal Medicine, Thumbay University Hospital, Ajman, ARE; 2 Internal Medicine, Hamad Medical Corporation, Doha, QAT

**Keywords:** learning disability, leptomeningeal angioma, mri, port-wine stain, seizures, sturge-weber syndrome

## Abstract

Sturge-Weber syndrome (SWS), also called encephalotrigeminal angiomatosis, is a congenital neurocutaneous disorder. It is characterized by facial and leptomeningeal angiomas as well as neurologic symptoms such as seizures and learning disabilities. We report a case of a 31-year-old male patient who presented with a history consistent with focal seizures. He was found to have a cutaneous angioma (port-wine stain) in the left temporoparietal region. CT brain revealed characteristic curvilinear calcification in the left tempoparietal cortex. The lack of tonic-clonic seizures and the pattern of his port-wine stain may have possibly delayed the diagnosis well into adulthood.

## Introduction

Sturge-Weber syndrome (SWS) was first described in 1860 by German physician and ophthalmologist, Rudolf Schirmer [1]. Today, it is recognized as a rare neurocutaneous disorder (affecting both the nervous system and the skin), typically diagnosed in childhood. However, atypical manifestations may lead to delays in diagnosis. We discuss a case of SWS in an adult patient whose diagnosis had been previously overlooked, potentially due to the unique manifestations of the patient’s epilepsy and intellectual disability. Our objective is to highlight the diagnostic pitfalls associated with SWS and compare the strengths and weaknesses of different radiological modalities and the possible redundancies among them, to underscore the potential reasons why clinicians may overlook a Sturge-Weber diagnosis.

## Case presentation

**Figure 1:**
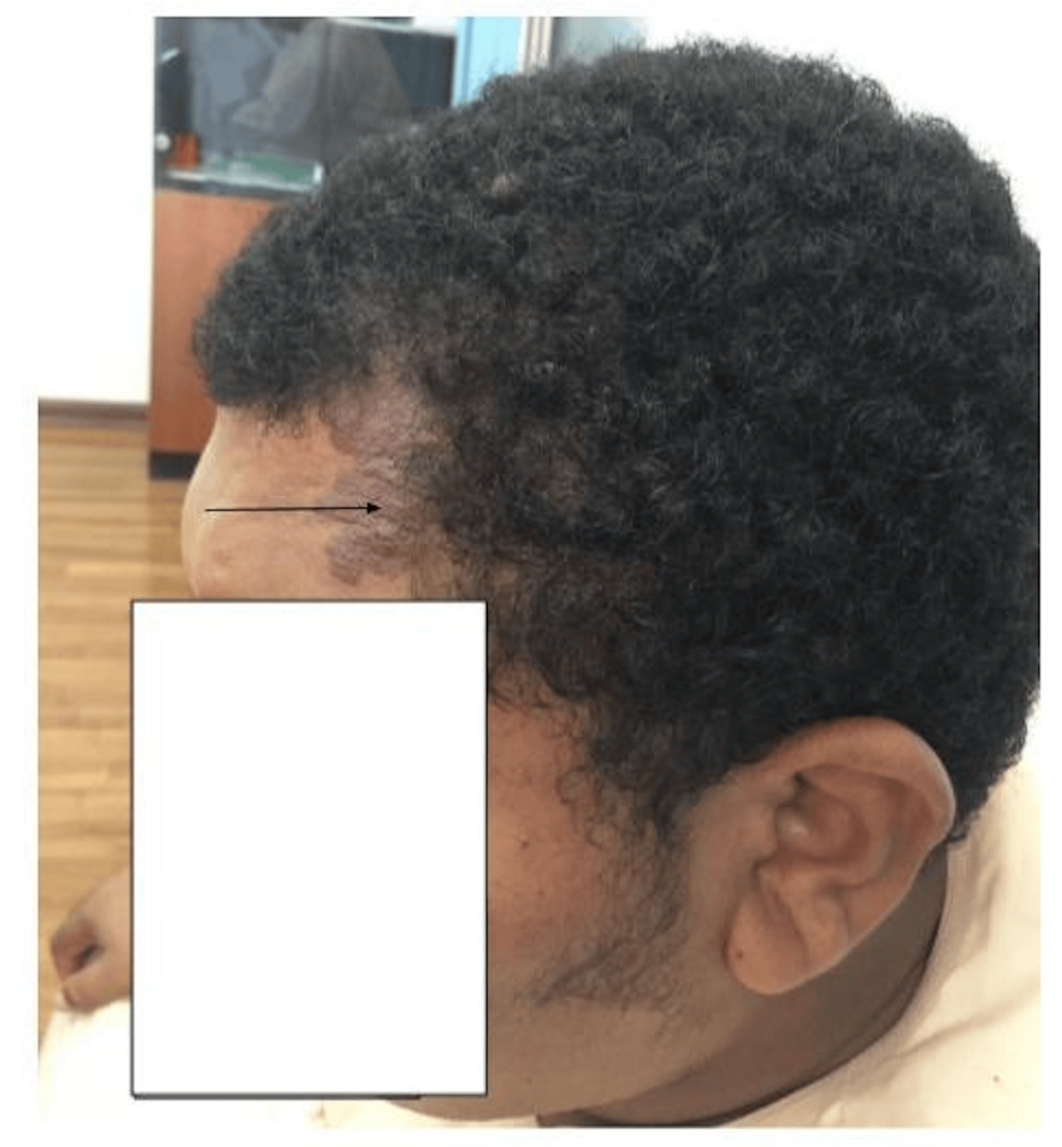
Cutaneous angioma (port-wine stain) involving the left side of the forehead and scalp (masked by scalp hairs)

A 31-year-old male patient presented to the internal medicine clinic with a two-month history of episodic uprolling of eyes and loss of consciousness. His past medical history was significant for morbid obesity (BMI: 41 kg/m²), type 2 diabetes mellitus, dyslipidemia, hypertension, and a history of learning difficulties since childhood, which had prevented him from enrolling in formal education. He did not have any involuntary limb movement. Such episodes lasted for about one to two minutes, and he subsequently regained consciousness. There was post-ictal confusion for two to three minutes. On examination, there was a cutaneous angioma (port-wine stain) involving the left forehead and left side of the scalp in the temporoparietal region (Figure 1).

**Figure 2:**
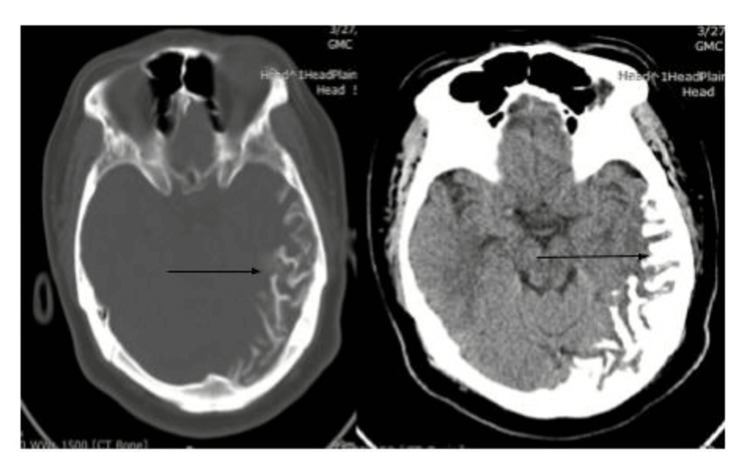
CT (brain Window) showing curvilinear leptomeningeal calcification is seen along left temporoparietal sulcal spaces as seen by the arrow CT: computed tomography

An awake electroencephalogram (EEG) was performed and was unremarkable. CT brain showed curvilinear cortical calcification along the left parietotemporal lobe with ill-defined hypodensity in the adjacent brain parenchyma (Figures 2a, 2b).

**Figure 3:**
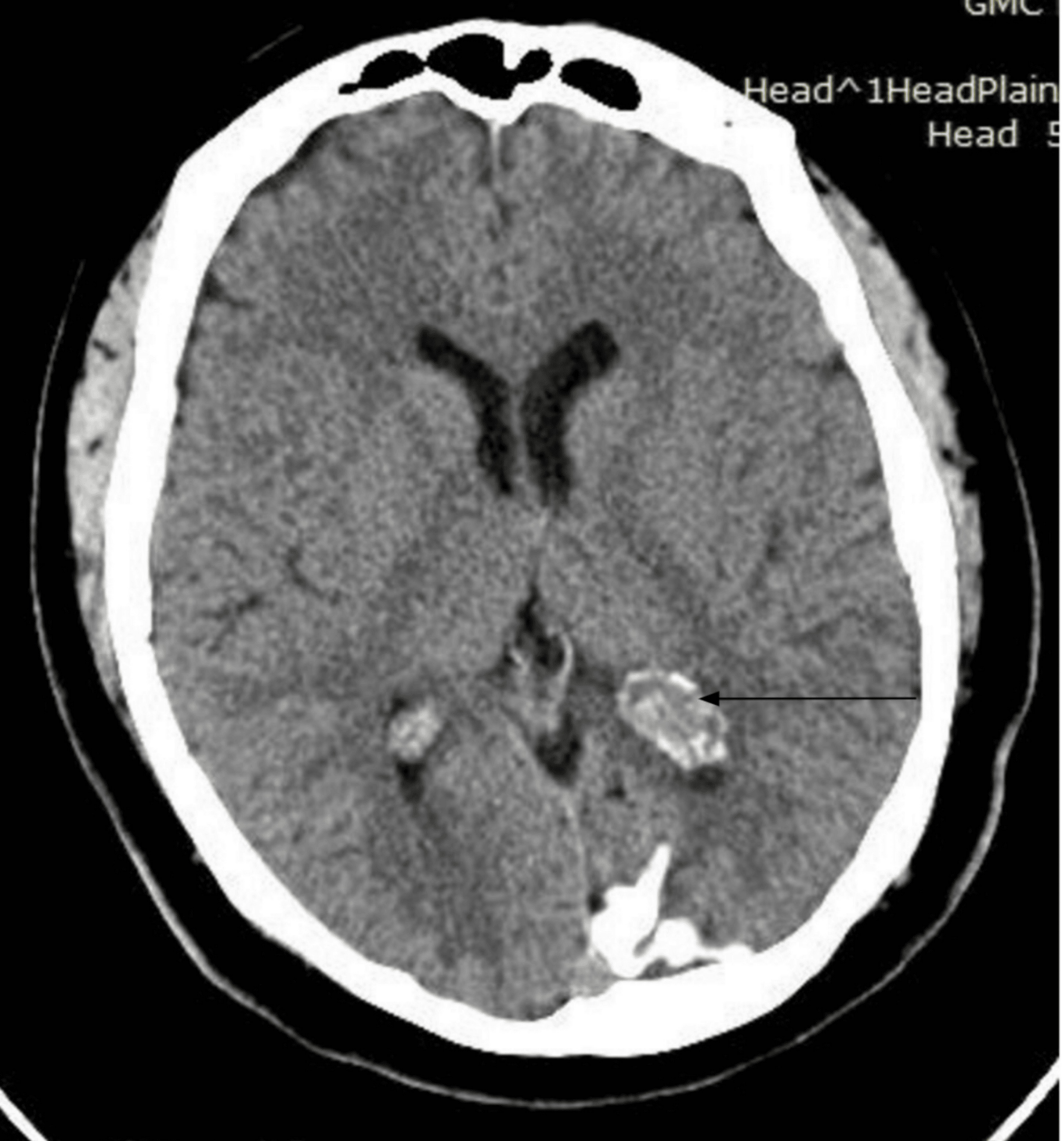
Left choroid plexus was prominent and showed calcification (marked by the arrow)

No edema or mass effect was observed. The left choroid plexus was prominent and showed calcification (Figure 3). The patient was treated with oral levetiracetam and lamotrigine. His anti-epileptic medication doses were gradually increased on subsequent clinic visits until he was seizure-free.

## Discussion

SWS is a neurocutaneous disorder characterized by neurological, ocular, dermatological, and radiological manifestations due to capillary malformations. Between 80-90% of SWS cases are attributed to a somatic, mosaic mutation in the GNAQ gene located on chromosome 9q21.2. This gene is responsible for the guanine-nucleotide binding protein G, which is involved in growth regulation. The mutation results in the proliferation of endothelial cells, resulting in capillary overgrowth. While the association of capillary and leptomeningeal malformations is not completely understood, theories have implicated the embryological proximity of the neural crest cells and the progenitors of the vasculature of the eye, face, and brain [1]. In terms of neurological manifestations, SWS may present with seizures, venous stroke and stroke-like episodes, and migraines [2]. Of note, 75% of patients with SWS experience their first seizure in the first year of life, and 86% of them before the age of two [3].

Neurological symptoms only occasionally present in adulthood, which is why it is uncommon for SWS to present past childhood [4]. However, our patient did not exhibit any convulsions until the age of 31. Furthermore, Comi showed that the seizures that occur in SWS are usually complex focal or partial seizures (with secondary generalization) [5]. First-line anti-epileptic medications in SWS are oxcarbazepine, topiramate, and levetiracetam [5]. Due to medication availability and physician familiarity, levetiracetam was prescribed with lamotrigine as an add-on therapy. EEG is an effective tool for the detection of epilepsy as well as cortical affection. EEG is more likely to detect subclinical seizures in the form of rhythm and voltage asymmetry than epileptiform abnormalities [6]. As seen in this case, intellectual and language impairments are common in patients with SWS [2]. The fact that our patient had no prior tonic-clonic or generalized seizure, coupled with the intellectual disability, which may have hindered his ability to communicate potential convulsions, may have further delayed his diagnosis.

Facial port-wine stains are a common, typically benign vascular malformation that may be associated with SWS in rare cases [7]. Port-wine stains with bilateral involvement of the upper eyelid portion of the trigeminal nerve's ophthalmic (V1) branch are associated with the highest risk of SWS [6,8,9]. The presence of a port-wine stain on the forehead and upper eyelid should prompt further neurological and ophthalmological evaluation [8]. The characteristics of port-wine stains are, therefore, an important consideration, especially when evaluating children. While our patient had unilateral involvement of the left upper forehead, the relatively small size and localized nature of the lesion may have contributed to his condition going undiagnosed.

The characteristic radiological feature of SWS is the presence of leptomeningeal vascular abnormalities, typically in the occipital lobe ipsilateral to the port-wine stains; however, other lobes may also show leptomeningeal abnormalities. Cortical calcification and cerebral atrophy may also be seen [10]. When comparing CT and MRI, CT is the more sensitive radiological investigation for detecting calcifications [10]. MRI, on the other hand, is more sensitive for detecting vascular abnormalities, the extent and degree of patency of the leptomeningeal malformation, and changes in the affected gray and white matter [11]. Hence, MRI with Gadolinium is the ideal modality for investigating SWS [12]. However, CT and MRI are complementary [10]. Even though calcifications were detected in this patient using CT, a limitation in our case was that an MRI was not performed due to financial constraints.

## Conclusions

In retrospect, an MRI should have been performed to complete the investigation of SWS in our patient. The particularities of presentation in our case (size and location of port-wine stain, lack of tonic-clonic seizures) might have contributed to the delay in diagnosis well into adulthood. This report demonstrates the importance of maintaining a high index of suspicion for SWS in patients presenting with intellectual disability and port-wine stain. Further research is required to establish a set of validated criteria for screening patients with port-wine stains for SWS, depending on the presentation of the patient (such as the location of the port-wine stain, neurological manifestations, and intellectual disabilities).
